# Programmed Cell Death-Ligand-1 expression in Bladder Schistosomal Squamous Cell Carcinoma – *There’s room for Immune Checkpoint Blockage?*

**DOI:** 10.3389/fimmu.2022.955000

**Published:** 2022-09-02

**Authors:** Ana C. Madureira

**Affiliations:** Faculty of Medicine, Eduardo Mondlane University, Maputo, Mozambique

**Keywords:** *S. haematobium*, PD-L1, Immune-Checkpoint-Blockage therapy, Bladder Squamous Cell Carcinoma, biomarkers

## Abstract

*Schistosoma haematobium*, the causative agent of urogenital schistosomiasis, is a carcinogen type 1 since 1994. It is strongly associated with bladder squamous-cell carcinoma in endemic regions, where it accounts for 53-69% of bladder-carcinoma cases. This histological subtype is associated with chronic inflammation being more aggressive and resistant to conventional chemo and radiotherapy. Immune-Checkpoint-Blockage (ICB) therapies targeting the Programmed-Cell-Death-Protein-1(PD-1)/Programmed-Cell-Death-Ligand-1(PD-L1) axis showed considerable success in treating advanced bladder urothelial carcinoma. PD-L1 is induced by inflammatory stimuli and expressed in immune and tumor cells. The binding of PD-L1 with PD-1 modulates immune response leading to T-cell exhaustion. PD-L1 presents in several isoforms and its expression is dynamic and can serve as a companion marker for patients’ eligibility, allowing the identification of positive tumors that are more likely to respond to ICB therapy. The high PD-L1 expression in bladder-urothelial-carcinoma and squamous-cell carcinoma may affect further ICB-therapy application and outcomes. In general, divergent histologies are ineligible for therapy. These treatments are expensive and prone to auto-immune side effects and resistance. Thus, biomarkers capable of predicting therapy response are needed. Also, the PD-L1 expression assessment still needs refinement. Studies focused on squamous cell differentiation associated with *S. haematobium* remain scarce. Furthermore, in low and middle-income-regions, where schistosomiasis is endemic, SCC biomarkers are needed. This mini-review provides an overview of the current literature regarding PD-L1 expression in bladder-squamous-cell-carcinoma and schistosomiasis. It aims to pinpoint future directions, controversies, challenges, and the importance of PD-L1 as a biomarker for diagnosis, disease aggressiveness, and ICB-therapy prognosis in bladder-schistosomal-squamous-cell carcinoma.

## Introduction

Schistosomiasis is a Neglected Tropical Disease (NTD) that affects over 240 million people worldwide, and 700 million are at risk of infection (1).

The clinical manifestations of the disease’Bs chronic and more severe stages are primarily due to immune reactions against Schistosoma eggs, the principal pathogenic agent, lodged in the tissues leading to granuloma formation (2). Granulomas are comprised of neutrophils, eosinophils, mononuclear cells, lymphocytes, macrophages, multinucleated giant cells, and fibroblasts (3). The granulomatous immune response is essentially coordinated by CD4+ T cells. Nevertheless, CD8+ T cells, B cells, and M2 macrophages also have a role (4, 5). Additionally, the overall egg-induced inflammatory immune response is Th2-biased (6). This polarization can be reverted by Praziquantel-intake as seen by the increase in pro-inflammatory egg-specific cytokine profile, namely Tumor-Necrosis-Factor-alfa (TNF-α), IL-6, Interferon-gamma (INF-γ), IL-12p70 and IL-23, after treatment (7).

The blood fluke *Schistosoma haematobium*, the causative agent of urogenital schistosomiasis, has been considered a carcinogen type 1 since 1994 (8). Moreover, it is strongly associated with bladder-squamous-cell-carcinoma (BSCC) in endemic regions. A Meta-Analysis reported that In Sub-Saharan-Africa it accounts for 53-69% of BC cases (9).

This histological subtype is associated with chronic inflammation being more aggressive and resistant to conventional chemo and radiotherapy (10–15). Immune-Checkpoint-Blockage (ICB) therapies targeting the axis Programmed-Cell-Death-Protein-1(PD-1)/Programmed-Cell-Death-Ligand-1(PD-L1) showed considerable success in treating several carcinomas, including the advanced bladder urothelial carcinoma (aBUC) presenting progressive disease and conventional therapies resistance (16, 17). This axis blockage may lead to complete restoration of the anti-tumor immune response improving disease outcomes (18, 19).

Programmed-Cell-Death-Ligand-1 is induced by inflammatory stimuli and expressed in the immune cells of the hematopoietic line, epithelial cells, and tumor cells, and in the latter, it aids immune evasion. This ligand binds to PD-1 during immune system modulation leading to T cell exhaustion (20). Its expression in the tumor microenvironment can be dynamic and present in several forms: membranar, exosomal, nucleic, and soluble. Such diversity can affect ICB therapy efficacy (21). Furthermore, soluble PD-L1 (sPD-L1) presents several variants, either derived from proteolytic cleavage or *via* matrix metalloproteases (MMPs) and A desintegrin and metalloproteases (ADAM), that are responsible for the shedding of exosomal mPD-L1, or by alternative splicing of PD-L1 pre-mRNA (21). sPD-L1 may also mediate immunosuppression or be responsible for ICB therapy resistance (21, 22).

The expression of mPD-L1 assessed by Immunohistochemistry (IHC) is a companion marker for patients’ eligibility for ICB treatment since it allows the identification of tumors that are more likely to be responsive (18, 19, 23). A higher expression is associated with better treatment outcomes. However, it was reported that lower/negative results don’t out rule successful ICB therapy (24–26), which may be associated with expression dynamics, tumor heterogeneity, different antibody clones, and staining platforms.

The performance and concordance of the established antibodies and staining platforms have been addressed in BUC and remain elusive (25, 27).

Program-Death-Ligand-1 is known to be overexpressed both in BUC with squamous differentiation and pure SCC (pSCC) and is associated with poorer disease outcomes (28).

This fact can have implications for further immune-therapy application and outcomes since divergent histologies are generally contra-indicated for ICB therapy since, despite mPD-L1 positivity, its overall incidence is low (14). Additionally, studies reported that variant and mixed histologies, including squamous differentiation, might not compromise ICB efficacy (29, 30). ICB treatments are expensive and prone to immune-related adverse events and resistance (31–33). Thus, biomarkers capable of predicting patient response are needed. In low and middle-income-regions, where schistosomiasis is endemic, SCC biomarkers are needed. Expression of sPD-L1 as BC biomarker has been previously approached (34), nevertheless needs further investigation in such settings.

Studies focused on the squamous-cell differentiation-associated or not with *S. haematobium* remain scarce. This mini-review provides an overview of the current literature regarding PD-L1 expression in BSCC and in Schistosomiasis. It aims to pinpoint possible future directions, controversies, and challenges as well as the importance of this ligand as a possible predictor biomarker for diagnosis, disease aggressiveness, and ICB-therapy success in schistosomal bladder-squamous-cell-carcinoma.

## Bladder Schistosomal Squamous Cell Carcinoma biomarkers – from *urogenital schistosomiasis to bladder carcinoma*

The exact mechanisms that drive *S. haematobium*-induced bladder carcinoma remain unveiled. The early diagnosis of this condition in a more non-invasive manner is eagerly pursued. Bernardo et al. (2015) used mass spectrometry and a proteomics approach to evaluate urine biomarkers and study molecular pathways associated with the development of bladder schistosomal – BSCC (BSSCC) (35). They reported a specific profile in patients with BSSCC consisting of higher expression of proteins involved in immunity (complement factor H, complement component 9), negative regulation of endopeptidase activity and inflammation (C-reactive-protein). These proteins are associated with inflammation (mediated by cytokines and chemokines) and signaling pathways related with bladder cancer proliferation (epidermal growth factor (EGF) and fibroblast growth factor (FGF) pathways). Additionally, prolonged inflammation may lead to DNA damage and mutations in the suppressor genes such as TP53 which leads to an overexpression of p53 (36). The study groups presenting only urogenital schistosomiasis (UGS), or BUC revealed a profile associated with microbicide activity such as oxidative stress and immune system related proteins and a profile associated with renal system process, sensory perception and gas and oxygen transport, respectively. The expression of S100 proteins was overlapping in the groups with BSSCC and BC. In patients with UGS, the upregulation of the nuclear factor kappa-light-chain-enhancer of activated B cells (NF-κB), pinpoints the Th2 biased response characteristic from UGS. A recent study, performed in murine models, that addressed the proteome tissue profile in the bladder, after egg-injection, also corroborated that this parasitosis may drive to urothelial hyperplasia and further bladder carcinoma. The study revealed a differentiated expression of several proteins associated with carcinogenic pathways, cellular activity and enhancement of immune inflammatory responses involved in granuloma formation, Th2 responses as well as reduced integrity needed for egg shedding (37). Amongst the proteins associated with malignancy, cell proliferation and cancer poor prognosis was disabled homolog 2 (Dab2) that was reported to be associated with epithelial-mesenchymal transition and tumor aggressiveness in BUC (38). Dab2 has a role in the canonical Tumor-Growth-Factor-beta (TGF-β) signaling in fibroblasts and immune tolerance, especially in regulatory-T-cells (Tregs) - mediated immunosuppression and toll-like receptor (TLR) suppression in antigen presenting cells (APCs) (39, 40). Other proteins associated with inflammation and tissue repair were also upregulated in bladder-egg-injected tissue, namely, complement component 8 (C8a), platelet endothelial cell adhesion molecule (Pecam1) and serine protease inhibitor A3N (Serpina3n) (37).

Several carcinoma biomarkers were investigated in patients with BSCC and BSSCC. Most of them determined by IHC. Badr et al. (2004) evaluated the expression of several tumor markers (p53, bcl2, HER2/neu, MIB-1) related with tumor-suppression, apoptosis inhibition and cell-proliferation. The study population consisted in 15 patients with BSSCC. They concluded that 87% of the cases presented a MIB-1 positive staining, followed by p53 (73%), HER2/neu (27%) and bcl2 (20%) (41). None was significantly associated with disease severity (higher stage and grade). Nevertheless, other authors reported that in patients with *S. haematobium* infection either with BUC or SCC, p53, MIB-1, Bcl-x and Bax were independent prognostic markers (42). Expression of cyclooxygenase (Cox-2), a marker of inflammation and angiogenesis was also associated with disease aggressiveness and poor-prognosis in patients with BSSCC but not in BSCC (43). Later, five biomarkers (Cox-2, p52, Bax, FGF-2, fibrinogen-growth-factor-receptor (FGFR)) were considered the best predictors for oncologic outcomes in a population with SCC where 80.8% presented UGS (44). Furthermore, a study performed in Sudan by Hassan et al. (2013) reported that the expression of Cox2 and Nitric oxide synthase (iNOS) could differentiate schistosomal from non-schistosomal BC, since both markers were highly expressed in patients with BSSCC (45). Nevertheless, these results were not entirely in agreement with another study that showed FGF-2 as the best predictor biomarker for disease outcomes in SCC. Its expression varied significantly according to tumor grade, presence of metastasis and lymphovascular invasion, but not with the presence of UGS (46). Additionally, the overexpression of FGR2 was positively correlated with higher expression of PD-L1 (47).

Were proposed several biomarkers to predict ICB therapy response. NOTCH homolog-4 (NOTCH4) has been correlated with a better response in several carcinomas including BUC (48). A recent study addressed FGFR3 mutations also as possible biomarkers for ICB therapy response. The study included BUC with variant histologies and reported that this marker was not correlated with PD-L1 expression or with pathological response to therapy (49). On the other hand, EFGR expression in bladder tissue has been correlated with squamous differentiation and predicted disease hyper-response after ICB treatment in patients with BUC (50).

Thus, the need of molecular biomarkers to differentiate BUC into molecular subtypes was highlighted given its importance and impact in ICB therapy outcomes. It is known that basal-like sub-types expressing higher levels of the cytokeratin’s CK5/6, CK14 and CD44 are associated with worse disease outcome (51). A study by Al-Sharaky et al. (2020) evaluated the expression of CK5, CK14 and CK20 in BUC and after stratification reported that CK5 was significantly associated S. *haematobium* infection in 81.1% of the cases. The sub-group CK5+/CK20- (basal) was only comprised by patients with SCC and schistosomiasis (52). Schistosomiasis was evaluated by microscopic observation of eggs in the bladder tissue which can temper the overall prevalence found (32.2%). More recently, Serag Eldien et al. (2021) reported a significant association between GATA3 lower expression and *S. haematobium* infection while CK5/6 expression was only associated with squamous cell differentiation (53). Patients with pSCC were excluded from the study. Nevertheless, *S. haematobium* infection associated carcinoma carried poor disease outcome (as short Progression Free Survival).

## PD-L1 in infections – *schistosomiasis case*

Infectious pathogens can modulate the host immune system to their benefit. Schistosoma spp are no different since they can polarize naïve-T-cells towards a Th1, Th2, and Treg phenotype depending on the antigen sampled during infection (54).

Studies performed *in vitro* and murine models reported the involvement of PD-L1 and several cells-subsets in Schistosoma infection-associated immune regulation according to the infectious stage of this parasitosis. Smith et al. (2004) demonstrated that *S. mansoni* warms can induce anergy in CD4+ and CD8+ T cells in the initial acute stages of infection *via* selective up-regulation of PD-L1 on the surface of Macrophages (Mϕ) (54). Additionally, the blockage of this ligand restored T-cell activation, thus demonstrating that up-regulated PD-L1 expression in Mϕ induces T-cell hypo-responsiveness granting *S. mansoni* worms the ability to subvert the immune-host response and to reach the egg-laying stage. Besides Macrophages, Dendritic Cells (DCs) were shown to regulate T- cell response either to Schistosome Egg Antigens (SEA) or towards cercaria *via* PD-L1 expression up-regulation. Klaver et al. (2015) showed that DCs stimulation with SEA induced the secretion of Transforming-Growth-Factor-beta (TGF- β) and the surface expression of the co-stimulatory molecules PD-L1 and OX40 ligand (OX40L) (55). PD-L1 and TGF-β induction was stimulated both on the mRNA and protein level. Furthermore, it was observed that the LPS-dependent cytokine induction in DCs was not affected by SEA-heat treatment since the expression of TNF-α, IL-6, and IL-12p70 remained impaired (55).

Later, Winkler et al. (2018) demonstrated that PD-L1 expression, along with PD-L2, IL-10, IL-6, and Macrophage-Inflammatory-Protein-1- alfa (MIP-1-α), was up-regulate on Dermal Dendritic Cells (DDCs) and in immature monocyte-derived DCs (moDCs) after infection by *S. mansoni* cercariae (56). The regulatory ability of extracellular vesicles (EVs), released at the schistosomula stage by *S. mansoni*, was shown to be driven by surface expression of PD-L1 on moDCs. Also, released EVs induced IL-10 and IL-12 overexpression by moDCs (57).

One knows that the induction of IL-10 in B regulatory cells is associated with immunosuppression during helminth infection (58, 59). Xiao et al. (2020) used murine models percutaneously infected with *S. japonicum* cercariae to investigate the B-cells profile and its role in CD4+ T-cells regulation, as well as the regulatory role of PD-L1 during infection (60). They observed that, after *in vitro* stimulation with SEA, B-cells assumed a regulatory phenotype *via* PD-L1 and CD5 up-regulation. Also, B-1a and Marginal-Zone-B-cells (MZB) percentages decreased, while the expression of IL-10, TGF- β, and INF-γ was up-regulated during acute and chronic infection. Additionally, during acute infection, B-cells could affect cytokine responses of CD4+ T-cells generating fewer effector memory cells and higher expression of Bcl-6. At the same time, PD-L1 expression resulted in a recovery of IL-4 production. It was also previously observed in B6 murine models that the expression of PD-L1 and PD-L2 was up-regulated during schistosome infection. Nevertheless, only PD-L2 was significantly reduced in the TLR-2^-^/^-^ model (61). This result emphasizes the latter’s role in the expression of PD-L2, such as the possible different immunological roles of PD-L1 and PD-L2.

A more recent study by Zhang et al. (2021) showed that Myeloid-Derived-Suppressor-Cells (MDSC) in *S. japonicum* infected murine models were able to regulate the T-follicular -helper cells (Tfh-cells) proliferation *via* PD-1/PD-L1 axis (62). The authors reported that SEA and SWA could induce the generation of Tfh-cells in which the PD-1 expression rises along with PD-L1 in MDSC.

The host-immune response towards *S. haematobium* eggs was also addressed in murine models. Bladder-wall egg injection replicated the immune-host response closely related to the human counterpart, including the Th2-biased response, the inflammatory granulomatous environment, fibrosis, egg excretion, and urinary tract morbidity. It was observed in murine models percutaneously infected with cercaria or/and bladder-wall injection an elevation of Th2-cytokines (IL-4, IL-13, and IL-5) at granuloma formation. The systemic profile revealed the same type of response (6). The only bladder cytokine affected by cercaria exposure was leptin (63).

Furthermore, IL-4 and IL-4R were associated with bladder pathogenesis and carcinogenesis in urogenital schistosomiasis (64). The correlation between schistosome antigens and BC was assessed. It was observed that higher antigen densities were correlated with squamous differentiation and with disease aggressiveness (65). Though, the PD-L1 expression wasn’t evaluated.

## PD-L1 expression in Bladder Squamous Cell Carcinoma – *a brief overview*

A PubMed search with the terms “urothelial carcinoma” AND “PD-L1” retrieved 587 results, while a search using the words “bladder squamous cell carcinoma AND PD-L1” retrieved 41 results. In the latter, only 4 matched the citation. The searches comprised results published between 2006 and 2016, respectively. The difference between results mirrors the lack of studies focused on PD-L1 expression on BC with squamous differentiation. Nevertheless, the number of publications increased within the last six years. The same string including the term “Schistosomiasis” retrieved one result. Most of the studies evaluated the inter-relationship between clinical-pathological features, the carcinoma subtype, and the expression of PD-L1 assessed by IHC (**Table 1**). The presence of *S. haematobium* was not evaluated possibly due to the study populations not being from endemic regions or to the retrospective design (**Table 1**). The only study that included urogenital schistosomiasis was performed in an Egyptian population (69). The PD-L1 expression was evaluated in tumor-micro-arrays (TMA) in a population with pSCC, from which 81.2% had a clinical indication of schistosomiasis. The positivity assessment relied on both immune cells (ICs) and tumour cells (TCs) scores (cut-off of >1%). Only the TCs score showed positivity. A cut-off of 5% was also performed with no positive results. The overall positive PD-L1 expression was 66.9% and the negative PD-L1 expression in the tumor was significantly associated with disease recurrence and cancer-specific mortality after adjusting for pathologic tumor stage, grade, lymph node involvement, and lymphovascular invasion. None of the established companion markers of ICB therapy outcome or staining platforms were used. It wasn’t stated how or when Schistosomiasis was diagnosed since the presence of eggs wasn’t reported.

**Table 1:** Summary of most pertinent publications were PD-L1 expression was addressed by IHC accordingly Squamous differentiation.

Author/Year/Country	Study Type	Sample Size	Tumor SubtypeN (%)			PD-L1 Positivity or higher positive score N (%)			Conclusions	Limitations
			pSCC	Mixed	UC	pSCC	Mixed	UC		
Pichler et al., 2017 (66) Austria	R	61	9 (14.8)	NA	48 (78.7)	NA	NA	10 (20.8)	Significantly high expression of PD-L1 was seen in the TC score of variant histologies including SCC (46.1%) when compared to pure Urothelial Carcinoma.	Limited number of samples with SCC. Was used a qualitative score for the quantification of PD-L1 (+) immune cells. PD-L1 expression was only reported in the overall mixed subtype.
Davick et al., 2018 (67) USA	R	165	23 (13.9)	NA	130 (78.8)	16 (70)	NA	52 (40)	PD-L1 expression varied according to grade and histological sub-type and was correlated with better OS. SCC showed a higher PD-L1 positivity when compared to UC (cases considered positive with TCs over 1%)	Was used TMA as sampling method for the staining Were only included cystectomy samples and lower stage tumors were under-represented. The scoring method to estimate the % of positive cells was semi-quantitative and can be observer dependent. Also, the number of cases with SCC was limited.
Udager et al., 2018 (68) USA	R	17	17 (100)	NA	NA	11 (64.7)	NA	NA	High PD-L1 expression was associated with basal-like molecular sub-types which may suggest successful ICB.	Small sample size. No comparative analysis was performed using other established staining platforms and scores.
Owyong et al., 2019 (69) Egypt	R	151	151 (100)	NA	NA	101 (66.8)	NA	NA	Most of the cases were previously diagnosed with urogenital Schistosomiasis (80.8%). These patients presented high PD-L1 positivity.	Weren’t reported the cases of BSCC with the presence of *S. haematobium* eggs. We’re not used established scoring criteria, companion antibodies or platforms for PD-L1 staining.
Reis et al., 2019 (25) USA	R	84	16 (19)	4(4.8)	NA	14 (88)	NA	NA	BCs with squamous differentiation presented higher PD-L1 positivity as well as higher TCs and ICs scores for all the criteria used in the study.	The study did not include pUC, instead compared the PD-L1 expression scores of the variant histologies with previously reported values.
Morsch et al., 2020 (70) Germany	R	108	63 (58.3)	45 (41.7)	NA	52 (82.5)	35 (77.7)	NA	There were no significant differences between the SCC and UC/UCC.	The study didn’t include pure UC samples for comparison.
Liu et al., 2020 (71) China	R	67	19 (28.4)	48 (71.6)	NA	13 (68.4)	28 (58.3)	NA	PD-L1 expression and high TILs were associated with poor disease outcomes in patients that underwent radical cystectomy without previous treatment.	Limited sample size. No established companion Abs, platforms or scores were used. Was only considered for PD-L1 positivity the TC score.
Gulinac et al., 2020 (72) France, Hungary	R	105	NA	5 (4.8)	91 (86.7)	2 (40)	NA	27 (30)	There aren’t statistically significant differences between PD-L1 expression accordingly histological sub-type. Higher PD-L1 expression was associated with age, gender, and higher tumor grade.	The short representativeness of samples with squamous differentiation comparing to pUC. No established companion Abs, platforms or scores were used.
Goderstsky et al., 2021 (73) USA	R	1478	135 (9.13)	217 (14.7)	1126 (76.2)	133 (86.9)	208 (53.5)	NA	Squamous cell differentiation either in BUCSD and pSCC cases is associated with worse OS and CSS. PD-L1 expression varies accordingly histologic subtype and may predict CSS in SCC patients. The PD-L1 expression didn’t varied according to any other demographic or clinical-pathological feature.	The use of TMA for the staining may not grant a representative staining due to tumor heterogeneity. The effect of ICB was not addressed. No comparative analysis was performed with UC.
Lee et al., 2021 (74) South Korea	R	219	NA	52 (23.7)	167 (76.3)	NA	28 (53.8)	102 (61.1)	No statistically significant differences were found between PD-L1 expression in histological subtypes.	The study didn’t include pSCC samples. Was used only one of the established companion Antibodies for the staining.

R, retrospective; NA, Non-Applicable, BUCSC, bladder urothelial carcinoma with squamous differentiation; Abs, antibodies

Pichler et al. (2017) evaluated the PD-L1 expression as a biomarker for disease outcome in patients with bladder carcinoma (BC) recurrence (66). A high PD-L1 expression in tumor cells (TCs) was associated with worse disease outcomes and was significantly higher in patients with variant histologies (46.2% vs. 20%), though pure or schistosomal-associated squamous-differentiation were not studied. Several molecular characteristics are associated with rare histologies, namely the frequency of biomarkers related to immunotherapy benefits, such as Tumor-Mutational-Burden (TMB), Microsatellite Instability (MSI), PD-L1 gene amplification, and IHC staining of the latter (75). Udager et al. (2018) explored the PD-L1 expression in a cohort of primary pure BSCCs and reported positive staining of 64.7% (68). There were no differences in the clinical-pathological features in this study amongst PD-L1 positive and negative samples. However, the association of PD-L1 expression with basal-like molecular subtypes was established. Also, cyclin-dependent-kinase-inhibitor-2A (CDKN2A) alteration was significantly higher in PD-L1-positive tumors. This was in concordance with Kim et al. (2020) that reported a PD-L1 high positivity strongly correlated with the Basal-Squamous-Like (BASQ) subtype irrespective of antibodies clones used (51).

Necchi et al. (2020) demonstrated that up to 5% and 1% of cases of pSCC and BUC, respectively, featured PD-L1 amplification (76). Remarkably, were no significative differences between these two carcinoma subtypes regarding PD-L1 staining since 2/3 of each study group showed positivity in TCs score and Tumor-Infiltrating-lymphocytes (TILs). Liu et al. (2020) evaluated disease outcomes and progression parameters against PD-L1 expression in non-schistosomal SCC pure and mixed (71). They reported an overall PD-L1 positive staining of 61.2%. The positive PD-L1 TCs score was associated with higher TILs density, independently of tumor histopathologic features and staging. The positivity was also associated with higher Progression-Free-Survival (PFS) and Overall Survival (OS). PD-L1 expression with higher TILs were independent protective factors affecting survival and PFS rate. Gordetsky et al. (2021) stated otherwise since squamous differentiation, in the mix and pure histologies, was associated with worse OS and Cancer-Specific Survival (CSS) and higher PD-L1 expression in TCs was a predictor for worse CSS in pSCC. In the IHC staining the authors used three clinically available antibodies. The positivity score varied accordingly histological subtype being higher in pSCC (73). These conclusions agree with a study performed by Lee et al. (2021). Nevertheless, PD-L1 expression was determined in tumor-infiltrating immune cells (ICs). The overall PD-L1 positivity of 59.4% was closely related to disease aggressiveness and shorter PFS. Furthermore, 53.8% of patients with Mixed BUC presented positive staining though there were no statistically significant differences between histological subtypes (74).

Reis et al. (2019) also assessed the expression of PD-L1 in BC patients with predominant or pure variant histologies using 3 of the antibodies clones available as companion markers for prognosis of ICB (cut-off value of 1 and 5%) and a combined positive score (CPS > 10%) (25). Amongst all of the divergent histologies evaluated, SCCs presented the highest positive score both for TCs and ICs for all the criteria established, though, as seen in Udager et al. (2019), the PD-L1 expression was higher in TCs from the periphery/invasive front of the tumor (68). Nonetheless, the TMB wasn’t determine since the basal molecular subtype is enriched with squamous differentiation. Instead, the authors assessed PD-L1 expression in TCs and ICs since these are the accepted host factors associated with ICB therapy’s improved outcome. A previous study, also using different established clones for PD-L1 IHC staining, and their established scores, reported that higher-grade tumors showed higher positivity for PD-L1, and SCC demonstrated PD-L1 positivity more frequently than BUC when using one of the clones (67). Nevertheless, the OS, according to histological subtype, wasn’t assessed.

A French and Bulgarian cohort study also demonstrated that PD-L1 positive expression was associated with higher tumor grade and stage. A higher CPS in BUC with squamous differentiation (72). Other studies addressed the PD-L1 expression in pSCC, demonstrating that overall, pSCC presented a higher PD-L1 expression when compared with tumors with mixed histology. Still, the difference wasn’t statistically significative. It was clear an inter-assay heterogeneity since both ICs and TCs scores varied accordingly to the Antibodies used (70). No comparative analyses were performed regarding pBUC.

Recently, the expression of sPD-L1 as a prognosis marker was examined in the scope of BC and correlated with disease aggressiveness and ICB therapy outcomes (77, 78). Vikerfors et al. (2021) evaluated sPD-L1 expression both in urine and serum of BC patients, from which 9.1% presented squamous-cell features (79). The serum levels weren’t significantly different between cases and controls. However, after stratification accordingly, disease aggressiveness, the levels diverged. On the other hand, urinary levels were significantly higher in cases compared to controls. Both were associated with disease aggressiveness, such as metastasis presence. Tosev et al. (2021) reported the same (34). However, the authors didn’t study the PD-L1 expression according to the histological subtype.

## Discussion – *controversies, challenges, and roads to take*

**Figure 1:**
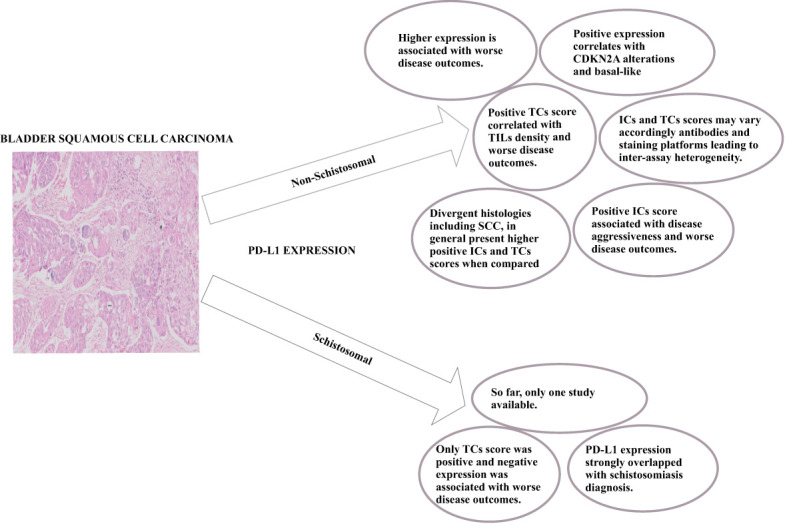
Overall key-points regarding PD-L1 expression in Non-Schistosomal and Schistosomal-Squamous-Cell-Carcinoma.

The high expression of PD-L1 is mainly associated with disease aggressiveness in several carcinomas, including BC with squamous differentiation (**Figure 1**). This histological subtype was also proposed as a biomarker for ICB-therapy outcomes and disease hyper-progression (50). PD-L1 expression assessment by IHC predicts disease outcomes and ICB therapy success (26). Nevertheless, despite its availability and cost-effectiveness, there are still pitfalls associated with the inter-assay heterogeneity due to different antibodies and staining platforms as well as different locations where PD-L1 is stained (TCs or ICs) (80). The biopsy can also be a factor for biased results, and challenging staining standardization since the tissue collected can be scarce, leading to misclassification (81). Furthermore, the antibodies available for the staining may not react to all PD-L1 isoforms (25).

The only study that addressed bladder schistosomal SCC didn’t use companion antibody clones and presented an unattainable ICs score (69). The authors performed the staining in TMA, which can temper the results given the shortness of tissue sampled. In this regard, further studies are needed since the expression of PD-L1 can be dynamic, as seen in studies that reported divergent expressions or weak correlations between mPD-L1 and sPD-L1 (77, 78). It was shown that mPD-L1 expression correlated with the expression of metalloproteinases in both tissue and serum (77, 82). Krafft et al. (2021) showed a positive correlation between serum MMP-7 and sPD-L1 in patients with BUC, while Pichler et al. (2021) demonstrated an inverse correlation between mPD-L1 and ADAM17 bladder tissue expression (77, 82).

The quantification of sPD-L1 has been associated with disease severity and worse prognosis in several carcinomas (81, 83–85); nevertheless, the studies remain scarce in BC. Urinary sPD-L1 in BC has been associated with disease aggressiveness and treatment outcomes, yet its expression, despite proper, may need further evaluation, namely, to assess the contribution of ICs (34). sPD-L1 can be of great value as a non-invasive form of disease assessment and diagnosis along with other immune mediators, especially in resource-limited settings where *S. haematobium* infection is endemic.

A study by Tetteh-Quarcoo et al. (2019) approached the early correlation between *S. haematobium* infection and carcinogenesis development *via* urinary cytological and wet-mount microscopic analysis. They observed several abnormalities, such as squamous cell metaplasia, inflammatory cells, and hyper-keratinosis, which may lead to a severe form of the disease, such as bladder cancer (86). Later, they showed that Praziquantel intake reduced those abnormalities (87). None of the studies investigated the host-immune response. Nevertheless, other studies reported that several cytokines are associated with *S. haematobium*-induced morbidity, namely TNF-α, IL-10, and IL-6 (88–90). Njaanake et al. (2014) reported a correlation between urinary levels of IL-6 with hematuria, heavy infection, and urinary tract pathology evaluated by ultrasonography (89). Interleukin-6 was associated with BC’s poor prognosis. An *in vitro* and *in vivo* study reported a correlation of urinary IL-6 with CD44+ expression in MIBC patients’ bladder tissue. Also, CD44+-cells expressed higher levels of PD-L1, which corroborates the role of this pleiotropic cytokine in invasiveness and immune suppression. The abrogation of IL-6 impaired CD44 expression and PD-L1 (91). Korac-Prlica et al. (2020) further referred to the importance of the combinatory immunotherapy approach since carcinogenesis, namely the progression to invasion and squamous differentiation, was impaired by the inhibition of the IL6/STAT3 pathway. Additionally, that fact sensitized BC to anti-PD-L1 immune therapy in an animal model with chemically induced MIBC (92).

In conclusion, the available data shows a correlation between Squamous-Cell differentiation and PD-L1 expression since in carcinomas with such differentiation it is higher. It is known that BUC presents a low PD-L1 (20-30%) expression when compared to other solid tumors (25). The PD-L1 expression reported in BSCC associated with Schistosomiasis was 66.8% which may render it as a plausible target for ICB. Nevertheless, further studies are needed taking into consideration the established criteria for ICB therapy. Furthermore, assess the correlation, role, and utility of cytokines and PD-L1 as disease and prognosis biomarkers, may be pertinent to better understand *S. haematobium*-associated-carcinogenesis, evaluate the benefit of ICB-combinatory-therapy, and overcome resistance and pernicious side-effects.

## Author contributions

AM confirms being the sole contributor for this work and has approved it for publication.

## Funding

AM is supported by the grant “Enhanced Advanced Biomedical Training in Mozambique” – D43TW010568-013UCSD2017SF – National Institutes of Health (NIH). This publication is of the entire responsibility of the author and does not necessarily reflect the official views of the funding agency.

## Acknowledgments

I’m thankful to the Anatomical-Pathology Service from Maputo Central Hospital and to Prof. Doctor Fabiola Fernandes and Dr. Lucília Lovane that kindly provided the Bladder Squamous Cell Carcinoma section image. I am also grateful to the Coordination of the PhD Program in Biosciences and Public Health by the support provided.

## Conflict of interest

The author declares that the research was conducted in the absence of any commercial or financial relationships that could be construed as a potential conflict of interest.

## Publisher’s note

All claims expressed in this article are solely those of the authors and do not necessarily represent those of their affiliated organizations, or those of the publisher, the editors and the reviewers. Any product that may be evaluated in this article, or claim that may be made by its manufacturer, is not guaranteed or endorsed by the publisher.
